# Comprehensive Investigation of Unmanned Aerial Vehicles (UAVs): An In-Depth Analysis of Avionics Systems

**DOI:** 10.3390/s24103064

**Published:** 2024-05-11

**Authors:** Khaled Osmani, Detlef Schulz

**Affiliations:** Department of Electrical Engineering, Helmut Schmidt University, 22043 Hamburg, Germany; alosmani.k@hsu-hh.de

**Keywords:** unmanned aerial vehicles, communication modules, embedded sensors, control algorithms, thermal imaging, obstacles avoidance

## Abstract

The evolving technologies regarding Unmanned Aerial Vehicles (UAVs) have led to their extended applicability in diverse domains, including surveillance, commerce, military, and smart electric grid monitoring. Modern UAV avionics enable precise aircraft operations through autonomous navigation, obstacle identification, and collision prevention. The structures of avionics are generally complex, and thorough hierarchies and intricate connections exist in between. For a comprehensive understanding of a UAV design, this paper aims to assess and critically review the purpose-classified electronics hardware inside UAVs, each with the corresponding performance metrics thoroughly analyzed. This review includes an exploration of different algorithms used for data processing, flight control, surveillance, navigation, protection, and communication. Consequently, this paper enriches the knowledge base of UAVs, offering an informative background on various UAV design processes, particularly those related to electric smart grid applications. As a future work recommendation, an actual relevant project is openly discussed.

## 1. Introduction

The evolution of Unmanned Aerial Vehicles (UAVs) is accompanied by instantaneous effects over many industries, thus reflecting a new era of telemetry, efficiency, and safety. At a first glance, agricultural surveying [1] and infrastructure inspection are alleviated by means of modern UAV technologies, hence reducing labor-intensive and risky tasks. From a different perspective, precision agriculture [2] is ameliorated through the sensors and cameras embedded in UAVs. Crop health can hence be better monitored, soil conditions accurately assessed, and resource utilization better optimized, contributing therefore to more sustainable farming practices [3]. Elsewhere, in military applications, UAVs with their highly versatile nature and reliability (i.e., remote control ability, durable flight periods, etc.), are found to be ideal for reconnaissance [4], border patrol [5], and disaster response [6]. Aside from agricultural- and military-based applications, environmental processes also benefit from UAVs, such that ecosystems can be remotely surveyed [7], wildlife can be dynamically tracked [8], and crucial data for conservation efforts and disaster management can be better gathered [9]. Forwardly, building inspection operations [10] and oil and gas infrastructure monitoring [11] can also be leveraged by means of UAVs for the commercial and industrial sectors [12]. For the part of mapping and surveying applications [13], terrains can be efficiently mapped, construction sites can be better monitored [14], and aid in urban planning [15] can be as well granted with high-resolution cameras and Light Detection And Ranging (LiDAR) sensors embedded in UAVs [16]. The ongoing innovation in UAV technologies, which is mainly based on advances in artificial intelligence [17], communication systems [18], and swarm technology [19], will eventually yield a continuous integration of UAVs into daily lives and industries [20]. It is of no doubt that UAVs are progressively evolving and widespread in diverse fields of application.

Despite the significant benefits and remarkable technological advancements associated with UAVs, their highly complex operational landscape implies various failure scenes [21], ranging from performance disruptions to risky mechanical failures [22]. Because of the sensitive hierarchies between the embedded electro-mechanical systems within UAVs, complexities arise from the integration of sensors [23], communication equipment [24], power systems, and others [25]. Firstly, UAVs can experience general system failures that are mainly linked to flight control programs [26]. Hence, maintenance challenges correspondingly emerge to ensure proper functioning. From another part, the inclusion of multiple electro-mechanical components yields increases in both the total weight of the drone and its power consumption, affecting its flight endurance [27]. Moreover, the interconnected system architecture induces a cascading failure effect when one component fails, potentially compromising the entire system [28]. Concerning the mechanical part, UAV’s continuous use and exposure to harsh operational conditions contribute to the wear-and-tear effect on different mechanical components [29]. A UAV’s structural frame and body can lose integrity, posing catastrophic consequences. Additionally, unavoidable vibrations [30] during flights induce resonance issues, successively leading to accelerated fatigue in the drone’s materials. In some applications, UAVs carry payloads, such as drop-off sensors or delivery packages [31], causing issues with their deployment or release mechanisms.

Besides mechanical failures, UAVs can be subjected to software bugs and glitches, leading to erroneous behaviors that affect their stability and performance [32]. For example, navigation errors are the result of inaccurate Global Positioning System (GPS) data [33] or misprocessed data in the UAV’s navigation algorithm [34], leading to deviations from the intended flight’s path. Regulation and compliance standards require a continuous update of UAV’s software to ensure continued adherence to legal and safety requirements [35], constituting a challenging chore. Where a UAV relies on remote communication systems for flight control and data transmission [36], any loss of communication (e.g., when UAVs operate beyond signal range limitations) poses a significant risk to the control and data transmission quality of the UAV (e.g., delayed responses and degraded data). Sensors inside a UAV, on the other hand, can be subject to failures [37], especially those that require precise calibration for accurate data interpretation [38]. Calibration errors or time-induced drifts lead to inaccurate readings (e.g., non-invasive current sensors reflect a false image over the actual current norm inside overhead electrical cables), hence compromising the performance of data collection [39]. In relation to the power systems inside a UAV, batteries (i.e., the main power source in a drone) can fail, with a sudden discharge or cell malfunctions [40]. Thus, the resulting loss of power can potentially limit the UAV’s flight time [41], and in the worst cases, causes the UAV to shut down and crash. On the other hand, environmental conditions [42] (i.e., weather) can impact UAV performance. For instance, proper UAV performance is affected by strong winds [43], heavy rain [44], and extreme temperatures as follows:

- Water-related challenges (i.e., rain) result in UAV operational limitations [45] as water can leak into the UAV, permanently damaging sensitive electronic components.
- Humidity: high levels of air humidity induce condensation and water accumulation inside a UAV.
- High temperatures: the performance of semiconductors inside a UAV is greatly affected by high temperatures.

With the mentioned challenges and obstacles objecting to proper UAV performance, the more detailed the knowledge about different UAV subsystems is, the better the overall design will be. In other terms, a detailed analysis of the hierarchies between the electro-mechanical components of a UAV, in addition to the sensory parts, with algorithms and data processing, serves as a predictive UAV maintenance scheme. Eventually, the performance of a UAV will be optimized by better decision-making (i.e., in the design part) and problem-solving (i.e., after a fault occurrence). In accordance, there are many drone-related literature reviews in the field, which serve in turn as informative backgrounds about different aspects of UAVs. In [46], the established review took only into consideration the interactions between a UAV and the payload (i.e., suspended loads), whereas in [47], the state-of-the-art focused mainly on drone detection and classification techniques. From another perspective, the review in [48] identifies current gaps in the application of UAVs for the creation of 3D models in the contexts of urban planning and historic monuments preservation. The review in [49] took into consideration the diverse possible applications of drones in healthcare applications, whereas in [50], the main focus was on the study of marine mammals (i.e., individual estimation, body conditions and biometrics, behavioral patterns, etc.). On the other hand, the review in [51] examined the drone-integrated Geographic Information System (GIS) in different fields, differently from the work in [52], which focused on UAVs’ potential to advance climate change research and monitoring. Although the work in [53] mainly accounted for the UAV remote sensing of crop species, it included some of the multispectral sensors used in such applications, thus enriching the informative background about UAVs. In a more general approach, the review in [54] considered a structured presentation of the recent trends in the UAV field, classifying them according to their flight characteristics, showcasing the potential areas for further development, and addressing the hardware/software within a UAV. In [55], the research considered an overview of anti-collision technologies for UAVs with the associated types of sensors, whereas in [56], the focus was on UAV threat models, with security and privacy aspects. Divergently, in [57], the focus was on search and rescue operations driven by UAVs, whereas in [58], the review examined the deployment of UAVs for monitoring and inspection in the construction industry. Concerning the You Only Look Once (YOLO) algorithm used for real-time detection and classification of multiple targets, the work in [59] investigated the integration of YOLO with UAV technology and the corresponding practical applications (e.g., engineering, transportation, automation, etc.). Given that path planning holds significance in the context of drone autonomy, the authors of [60] reviewed the environmental representation as well as the path generation techniques for drones. Considering the potential of UAVs in warehouse management, a systematic literature review was conducted in [61], enriching the background of knowledge about the obstacles versus the adoption of UAVs in warehouse handling, unlike in [62], where the review primarily focused on the role of drones in flood management. Different UAV platforms for autonomous applications are reviewed in [63], which also presented the state-of-art and estimation techniques for UAVs, in addition to their correspondent flight phases.

As can be noted from the literature survey encompassing the modern reviews in [46,47,48,49,50,51,52,53,54,55,56,57,58,59,60,61,62,63], the majority of these articles actually focus on the UAV domain of usage and application fields. In other terms, there exist no reviews about the drones themselves, involving different architectures, structures, avionics, and software-to-hardware branches (i.e., the results of algorithms over the behavioral mechanism of sensors). Forwardly, as a matter of proof, and despite the extended application fields for UAVs such as concentrating solar thermal system planning and operation [64], intrusion detection [65], forest ecology [66], forensic civil engineering [67], plant-scale growth estimation [68], and photovoltaic plant diagnosis and maintenance [69,70], the available related reviews provide a less detailed informative background about the realm of UAVs. To address this issue, this paper aims to critically review and assess the different UAV facets, decomposed into five major sets including flight control and navigation, communication and remote control, computing and data management, sensing and monitoring, and power and energy management. The intended structure of this review aims to establish an informative background concerning the UAV compositional realm, instead of possible field applications, for UAV design processes.

The rest of this paper is structured as follows: Section 2 presents the research methodology used to obtain data, Section 3 outlines a general UAV operational overview in which a comprehensive synopsis of UAV operations is presented, Section 4 elucidates the actual review of different sets (i.e., subsystems) within a UAV, Section 5 discusses the obtained results, Section 6 highlights an actual UAV-based future project design with a corresponding link to this review, and finally, in Section 7, conclusions are derived.

## 2. Research Methodology

Because of UAVs’ widespread applications in various industries, as indicated in the Introduction, extensive literature in this field has arisen. This fact in turn creates a challenge while conducting a thorough review of UAVs’ internal structures and composers. To address this challenge, and to better guide interested audiences through the different aspects of UAVs, the systematic approach adopted here consisted of dividing a UAV into five main sets as follows:

1. Control: This set includes navigation systems, flight control, autopilot, collision avoidance, target tracking, fail-safe, motor speed, and other related systems that are dedicated to managing and directing a UAV’s flight.
2. Computing: This set includes the computational elements including data processors, onboard computers, data loggers, and all computing platforms responsible for the execution of different algorithms.
3. Communication: This set includes the information exchange between a UAV and external parties (i.e., for remote control options) performed through different communication modules (e.g., Bluetooth, Wi-Fi, Long Range (LoRa) modules, etc.).
4. Sensory: This set includes data captured from internal (e.g., UAV’s power consumption and temperature), as well as external (e.g., altitude, pressure, and wind speed) environments held through UAV-embedded sensors.
5. Power: This set includes the energy sources (e.g., battery/solar cell drives), power distribution, and power management systems with the related circuitry to provide UAVs with optimal power for proper overall functionality.

Technically speaking, this research began by searching for relevant keywords, such as flight control in UAVs, avionics integration, UAV autonomous navigation, different power source types for launching UAVs, communication modules in UAVs, and others. In the first step, the corresponding results formed a large database, which was decomposed later according to the previously mentioned five main sets. Concerning the aim to inform readers about different aspects of UAVs, the conducted structure was developed in the form of relative answers to the following research questions (RQs):

RQ1: What are the different subsystems within a UAV?
RQ2: Is there any hierarchy between the different subsystems?
RQ3: Is there any integration between the subsystems?
RQ4: How can UAV reliability be enhanced by means of multiple sensory systems?
RQ5: What are the programming languages for different computing systems?
RQ6: What is the relationship between sensory and computing systems?
RQ7: How is the interdependence between subsystems managed?
RQ8: What are the standard UAV communication protocols?
RQ9: How can fail-safe be ensured in emergencies?
RQ10: How can limits be set for motor speed and UAV maximal altitude?

The answers to the raised questions consequently shaped the general overview of this manuscript, allowing thorough knowledge to be gained about the various facets of UAVs. The methodologies inside each set are mutually compared and assessed. Ultimately, concerning the resultant tabulated performance and other metric data for each set, the relevant discussed project highlights the contribution of this paper’s outcomes to the body of knowledge of UAVs.

## 3. UAV Operational Overview

**Figure 1:**
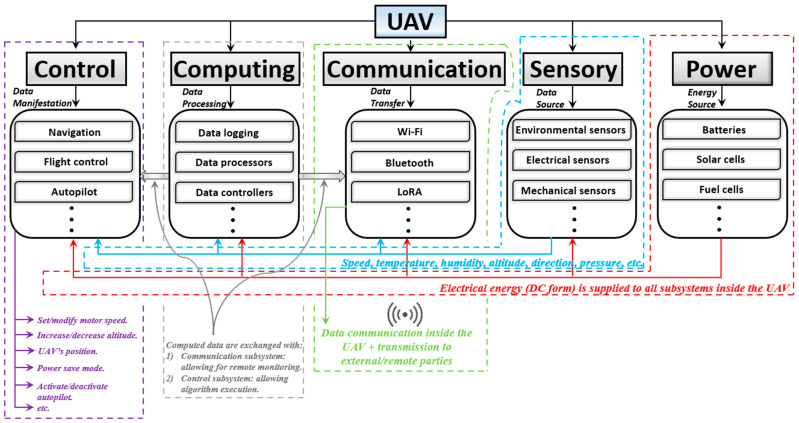
General overview of a UAV’s internal subsystems with the corresponding links.

Each of the main sets emphasizes a function (or a set of functions) as its collaborative job with respect to other sets, which are together aimed at achieving the accurate, stable, and successful flight of a UAV [71]. The key to a general understanding of UAV technologies is to establish a relationship between the five main sets according to two parameters, as discussed in this review, including hierarchy [72] and integration [73]. Figure 1 presents an overview of the hierarchy and integration between the five sets.

Additionally, a hierarchy exists between the composers of the same set. For instance, the commands from the autopilot system override the flight control and lead the navigation system [74]. The collision avoidance system utilizes data from the sensory subsystem as well as from the navigation system [75]. Higher prioritized hierarchies [76] (e.g., fail-safe) override and modify commands and data from other systems, where, for example, all sensory data would be instantaneously blocked to reduce computational efforts on the central processor and ensure that the UAV shuts down safely. As for hierarchies between the composers of different sets, the data processors on the first hand receive inputs from sensory subsystems, and sometimes from the control subsystem, for real-time processing [77]. The equipped computers on the UAV manage and coordinate tasks across all subsystems by executing flight control algorithms based on the processed data [78]. Data loggers, on the other hand, save different input data for further investigation during the same flight (i.e., metaheuristic analysis-based flight modification) or offline (when the UAV is not on a mission) [79]. The UAV’s health and performance indices are captured by internal sensors, thus providing data to both the control and computing subsystems, where external sensors share environmental data with the corresponding computing subsystem for decision-making [80]. As shown in Figure 1, the control subsystem establishes real-time decision-making and executes flight control algorithms when integrated with the computing subsystems (i.e., based on the processed data output from sensors). In turn, the communication system, aside from facilitating interaction/integration among all subsystems, enables real-time command reception/execution when integrated with the control subsystem. From the different perspective of integration/hierarchies, a UAV’s technology may implement mechanical redundancy systems [81] (i.e., to ensure reliability in the case of failures), encryption [82] (i.e., for secure data exchange/communication), and payload swing suppressor [83], which can act as standalone subsystems.

## 4. Avionics Assessment

As indicated in the Introduction, this review consists of five main subsystems of UAVs (control, computing, communication, sensory, and power). Concerning the control review, it includes different schemes for navigation (strategy, path planning/obstacle avoidance, localization), target tracking, and payload integration. The computing-based review encapsulates the different single-board computers, as well as the system on modules, used for data processing in UAVs. The communication review presents the possible communication protocols along with their relative modules, which can be UAV-embedded. Sensors are mainly classified as environmental-, vision-, and position-based, with each set including different technically reviewed physical sensors. Finally, the power review includes different sources that can be utilized to power up UAVs.

### 4.1. Control

The control subsystem inside a UAV manages and directs its flight operations, with its decision-making process relying on sensory data. Its primary purpose is to exercise precise command over the UAV’s navigation, stabilization, movements, positioning, balancing, take-off, and landing. In other words, the control subsystem acts as the brain and steering mechanism of a UAV. As can be concluded from Figure 1, the computing subsystem interacts with the control subsystem by providing the necessary algorithms, whereas the communication subsystem allows dynamic adjustments through real-time data exchange.

#### 4.1.1. Navigation

Assuming that a UAV has its path already determined (i.e., concerning its mission, purpose of usage, etc.), its navigation involves the real-time process of guiding it along that path. It can therefore be perceived as the constant monitoring of the UAV’s position, velocity, and orientation with respect to the planned trajectory. As a short general description, under the control subset of a UAV, navigation refers to the ongoing execution and adjustments of the UAV’s movement along its planned path. Other related reviews in the field mainly classify UAV navigation techniques as either indoor (i.e., used in closed areas where GPS signals, for example, can be weak or unavailable), or outdoor environments (i.e., for aerial surveying, crop monitoring, and rescue missions). Indoor navigation methods, on the one hand, can be achieved using a packet loss-based approach [84]. In GPS-denied environments, a drone is capable of estimating its current position based on the wireless packet loss from a reference point packet transmission. Specifically for indoor corridor environments, a UAV can be navigated through a Convolutional Neural Network (CNN), resulting from the combination of ResNet and DenseNet networks [85]. The CNN analyses the images captured by the UAV’s monocular camera and estimates the position/orientation of the UAV relative to the environment. A precise indoor UAV location estimation can also be obtained by means of ultrasonic acoustic signals with a three-stage localization scheme [86]. Outdoor UAV navigation methods, on the other hand, mainly rely on GPS [87], Inertial Navigation System (INS) [88], Global Navigation Satellite System (GNSS) [89], integrated INS/GNSS [90], and others [91]. This manuscript, however, conducts another approach for reviewing UAV navigation techniques, namely, classified as navigation strategy, path planning/obstacle avoiding, and localization.

*Strategy*

(a) Vision-based techniques

In general, vision-based navigation techniques have evolved from the need for proper UAV navigation in GPS-challenging (i.e., indoor) environments and for collaborative navigations for swarms of UAVs. This is also the case when two UAVs exist in contradictory environments, where one UAV operates under a good GPS coverage, unlike the other. In such circumstances, the navigation can be held using a father–son information exchange [92]. By means of line-of-sight (LOS) communication between the father and son UAVs, navigation information can be exchanged and acknowledged to the son UAV through the father UAV installed onboard camera, thus providing related data such as the Body Reference Frame (BRF) [92]. Vision-based navigation systems for UAVs can also act as backup for signal-enabled navigation methods (e.g., GPS, INS, etc.) for cases of jamming and drift errors, where the pre-obtained latitude and longitude data are utilized after their integration with the Google static map Application Programming Interface (API). UAV position estimation is hence acknowledged using matching techniques, such as normalized cross-correlation with prior edge extraction [93]. Map generation during navigation is another effective vision-based technique to guide a UAV under undesirable weather conditions (e.g., rain, storms, etc.) [94]. For instance, a high-resolution depth map can be developed by means of segmented optical flow from two successive frames, yielding a dense map picture reconstruction by optimizing a convex algorithm [95]. On the other hand, by means of a stereo visual-inertial (SLAM), with a two-step refreshing approach of an awareness map, the occupancy state (i.e., positioning cells in the map) is then updated, thus allowing for a visualization-enabled UAV navigation [96].

Vision-based navigation for UAVs is a popular trend in the field of UAV technology, mainly because of its sequential working mechanism, utilizing real-time target, environmental, and other data as the basic input to reveal a UAV’s instantaneous position after data processing through an onboard computer [97,98,99,100]. Overall, this set of navigation strategies relies on visual clues, thus offering dynamic spatial awareness for the UAV to build decisions upon. For instance, a UAV can navigate through unstructured terrains with precision because of real-time visual data interpretation. The BRF, on the other hand, ensures an accurate maneuvering of the UAV, as is the case with map construction (i.e., through harnessed images). From another perspective, vision-based strategies, mainly dependent on graphics, are susceptible to poor lighting conditions and visual clutter, leading unfortunately to errors in navigation. Table 1 encapsulates the pros/cons of vision-based UAV navigation methods, along with the incorporated challenges and different considerations.

(b) Artificial Intelligence (AI)-based techniques

Unlike vision-based navigation techniques, Artificial Intelligence (AI) contributes toward better autonomy in UAV navigation [101], mainly by allowing drones to learn from their surroundings [102]. From another perspective, AI allows for automatic feature extraction while better managing onboard resources, which differentiates it from traditional cognitive algorithms [103]. Concerning UAV navigation, AI algorithms are characterized in this manuscript according to their paradigm as follows: AI algorithms that involve mathematical model formulation to find the best solution to a given problem by relying on predefined rules and objectives to guide the UAV are grouped in the first set (i.e., mathematical optimization) [104]. The other set emphasizes a paradigm that trains the models to make better decisions in UAV navigation (i.e., performance is evolved over time based on training data and gained experience) [105].

(b.1) Mathematical optimization

For any given non-deterministic polynomial complex problem (i.e., the navigation path of a UAV), mathematical optimization-based AI approaches achieve near-optimal solutions [106]. The most dominant algorithms in this field are Particle Swarm Optimization (PSO) [107], Ant Colony Optimization (ACO) [108], Genetic Algorithm (GA) [109], Differential Evolution (DE) [110], and Gray Wolf Optimization (GWO) [111], as assessed in Table 2 and defined below:

- PSO: the optimal path for particles (i.e., drones with a swarm) can be attained by means of a competition strategy-based PSO, after comparison between the current global path with respect to other global candidates [112].
- ACO: the premature convergence of a single-colony ACO algorithm can be overcome using multi-colony ACO, where multiple UAV groups search for the optimal routes to the destination [113].
- GA: the 3D position of a UAV is encoded into a chromosome which in turn contains information about the UAV’s position/motion (e.g., acceleration, rate of the climbing angle, rate of the heading angle, etc.). The present-time 3D coordinates are obtained from the chromosome decoding and then evaluated by a fitness function. Eventually, path selection and information loss/exchange are referred to genetic operations [114].
- DE: in the case of a disaster (i.e., the navigation becomes harder), a constraint DE converges toward the optimum UAV route by selecting the high fitness values and minimum constraint violations among all probable traveling points [115].
- GWO: for fast convergence and efficient environmental exploitation, the conventional GWO can be hybridized with other algorithms (e.g., modified symbiotic organisms search), eventually yielding better UAV path navigation [116].

Each of the algorithms presented in Table 2 is nature-inspired, and they all aim to efficiently explore the space of available solutions to achieve the best/optimal solution (i.e., in this case, the best navigation route). Additionally, they utilize a population-based approach, such that multiple candidate individuals (e.g., swarms in the case of PSO) are iteratively improved. Optimal performance (i.e., finding the optimal route) is eventually achieved by means of parameter tuning. In comparison with vision-based navigation techniques, such algorithms require no image processing, thus implying no graphics complexities (e.g., resolution, light conditions, etc.). The shared drawback among these algorithms is the slow and/or premature convergence, reflecting reduced navigation accuracy.

(b.2) Training models

Representing model-based AI algorithms, training models aim to achieve near-optimal solutions (i.e., best route/path for a UAV) by means of self-training and to learn how to perform continuously better decision-making [117]. On the one hand, Reinforcement Learning (RL) [118,119,120] allows UAVs to learn about the environment, yielding optimum navigation. Because an agent and the environment represent the fundamental component of RL, through interaction with the latter, an optimum path can be chosen by the agent (i.e., UAV) using a Markov decision process [121]. From another perspective, based on the reward-to-target compensation system (i.e., the closer the UAV to the target, the more reward given by the environment), a Q-learning algorithm navigates an indoor UAV through the control of a Proportional Integral Derivative (PID) controller [122]. Amid the Q-learning algorithm context, Deep Reinforcement Learning (DRL) can be utilized for UAV navigation by using Q-values, such that the Q-table is replaced with a Deep Neural Network (DNN), hence offering better scalability [123]. Instead of performing repetitive manual calculations, as is the case with the Q-table, the DNN can make predictions and visualization the same way the human brain does [124]. Deep Learning (DL), on the other hand, with its different types such as Fully connected Neural Networks (FNNs) [125] and Convolutional Neural Networks (CNNs) [126], helps in autonomous UAV navigation under harsh environments by only utilizing the DNN part of the DRL. For instance, a UAV can be navigated through DNN by means of an image augmentation method [127], as well as via real-time photos and CNNs [128].

Despite the numerous advantages offered by training model-based AI in the field of autonomous UAV navigation, such techniques require long training time and large computational power, in addition to presenting high complexity in algorithm implementation, the need for extended information updates, and slow adaptation to new environments [129,130]. Generally, machine learning algorithms, as in the stated examples of FNNs and CNNs, are restricted by computational power. This is mainly due to their functional mechanism, which resembles heuristic search algorithms. In other terms, such training models use computers to search and resolve the algorithm automatically through massive trial-and-error. The algorithm is hence modeled using parametric optimization, beginning with the framework, equations, and structure defined with initial parameters. The end goal in these training models is to find the optimized parameters, through which the problem (in this case, finding the best route for a UAV) can be solved. Therefore, smarter training models (i.e., with more parameters to achieve a sharper convergence toward an optimal solution) require more computational power. For such reasons, and in order to reduce complexities in the hardware (e.g., GPU) implementation of these models, modern research aims to optimize DL models by reducing their energy consumption and memory requirements. By means of parameter quantization and pruning, network architecture search, knowledge distillation, and compressed convolutional filters, the high computational power of CNNs and FNNs can be reduced [131].

*Path planning/obstacle avoidance*

Navigation strategies encompass the overall approach guiding the UAV, mainly including the physical execution of algorithms through actuators (i.e., motors). It is unlike path planning and obstacle avoidance, which focus on determining the optimal trajectory while reacting to the real-time obstacles encountered along the planned path. In other terms, path planning/obstacle avoidance considers a feasible trajectory for the UAV to follow between start and end points, ensuring a smooth path while detecting and avoiding obstacles in the airspace. For the mentioned purpose, on the one hand, there exist passive and active sample-based algorithms, such as rapidly exploring random graphs [132], Probabilistic Road Maps (PRMs) [133], Rapidly exploring Random Trees (RRTs) [134], and Dynamic Domain RRT [135]. In addition, obstacles can be momentarily avoided (i.e., collision-free path) in collaboration with the RRT algorithm [136]. Other path-planning algorithms can also be mathematically based (e.g., Mixed-Integer Linear Programming (MILP) [136], integer programming [137], and non-linear programming [138]). Different path planning algorithms can also be fused together to obtain better convergence accuracy and to overcome the drawbacks of each algorithm. For example, in [139], a 3D PRM is used to form the roadmap, combined with the A* node-based optimal algorithm, to find the optimal obstacle-free path. From a different perspective, path planning can be bio-inspired, as such algorithms require less learning. For instance, the Bio-Inspired Neural Network (BINN) and Sparrow Search Algorithm (SSA) in [140] scan the flight environment, smoothing it for further safe surface obtainment. By means of SSA, the nodes are determined with the lowest comprehensive cost, hence achieving dynamic obstacle avoidance. Table 3 lists different sets of path planning and obstacle avoidance algorithms.

*Localization*

**Figure 2:**
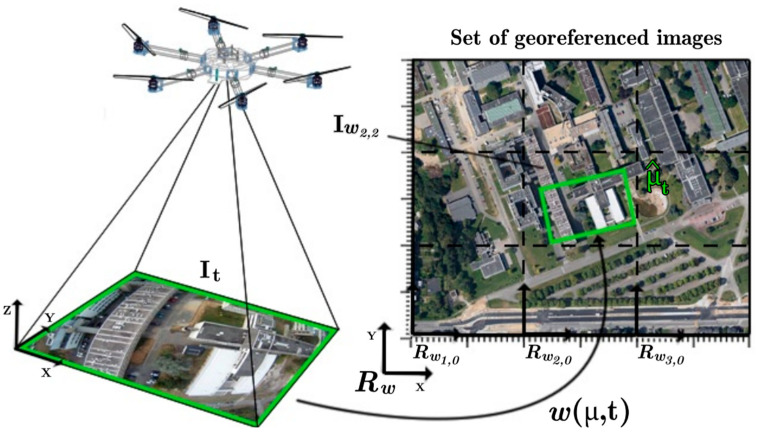
UAV localization through a set of georeferenced images [154].

The localization of a UAV is the process of determining its position in a given environment, involving the obtainment of accurate information about the UAV’s spatial coordinates (i.e., latitude, longitude, orientation, etc.). In relation to other navigation hierarchies (e.g., path planning), it enables a precise execution of the planned paths and strategies [141,142,143,144,145]. Concerning vision-based UAV localization techniques, they can be characterized according to two main sets as follows: Relative Visual Localization (RVL) [146,147] and Absolute Visual Localization (AVL) [148]. Among RVL methods, the popular Visual Odometry (VO)-based techniques [149] analyze the difference in “egomotion” by comparing between current and previous frames while performing with Optical Flow (OF) analysis [150]. Generally, all RVL methods suffer from drift over time [151], ignited by the usage of recursive estimations in order to formulate new estimations. Because of this, AVL methods have evolved in the field of UAV localization, mainly because of their inherent immunity against drift over time. Unlike the working mechanisms for UAV localization in RVL methods, AVL localizes a UAV by means of reference data, which are mainly composed of precisely georeferenced aerial images [152]. For example, a UAV’s absolute position can be acquired by means of normalized cross-correlation [153], as well as via a Mutual Information (MI)-based dense approach, as shown in Figure 2 [154].

The global reference map in Figure 2 is constructed from a mosaic of georeferenced images, such that an “sRt” model (i.e., accounts for the 3D motions) is produced for usage after an assumption of a planar ground and a UAV image that is parallel to it, defined as in (1).

(1) $$w\left(X_{t},\mu \right)=sR_{2d}x_{t}+t_{2d}$$

where $R_{2d}\;$ represents a 2D rotation, s  is a scale factor, $x_{t}\;$ is a point in space, and $t_{2d}\;$ is a 2D translation. The MI function’s maximum is found using Newton’s optimization warped in the “sRt” model with respect to the global reference map. Results indicate small relative mean squared errors in the localization data [154]. AVL methods for UAV localization overcome the drawbacks of GNSS-based localization methods, such as reception issues [155], spoofing attacks [156], and signal degradation [157].

#### 4.1.2. Target Tracking

Within a UAV’s field of vision, target tracking refers to its process involving the identification and localization of certain objects or points of interest [158]. Such a procedure begins with data capturing (i.e., images of the surroundings) [159] through the embedded cameras. Obtained data can then be pre-processed in order to ameliorate its quality, therefore rendering the target tracking algorithm more accurate [160]. The target tracking algorithm afterward identifies objects of interest in the processed data, thus providing class probabilities and bounding box coordinates [161]. After refining the results through filtering techniques [162], the UAV’s path control can be adjusted accordingly [163]. In this context, regression-based approaches aim to compute the object’s correlative and class probability, including YOLO [164], YOLOv2 [165], YOLOv3 [166], YOLOv4 [167], and YOLOv5 [168]. On one hand, YOLO aims to detect small objects in real time by identification in image frames. The input image (i.e., taken by the UAV’s camera) is divided into a grid, where, for each of the grid cells, the algorithm predicts the class probabilities, bounding boxes, and object scores. Bounding boxes represent the potential locations of objects inside the cell, and class probabilities are then assigned to each bounding box (i.e., the likelihood of an object belonging to a particular class such as a car, person, etc.). At the last stage of the YOLO algorithm, the certainty regarding the object inside a box is calculated after multiplying the class probability with the confidence score. Despite its real-time image processing capability, YOLO may face challenges in adapting to dynamic environments, localizing small objects, and handling overlapping objects [169].

With the drawbacks of YOLO, YOLOv2 represents an updated version with enhanced bounding box predictions through the implementation of anchor boxes. Additionally, YOLOv2 can handle more object categories because of its incorporated hierarchical classification with enabled joint training on multiple datasets. It can be therefore stated that YOLOv2 detects more diverse object classes than YOLO with an improved overall accuracy. Still, YOLOv2 suffers from overlapping object suppression difficulties [170]. Successively, as an improvement of YOLOv2, YOLOv3, consists of a hybrid architecture composed of Darknet-53 [171] and ResNet [172]. It detects multi-scale objects via a feature pyramid and integrates the regression of the bounding box, class prediction, and score calculation in a single forward pass. Despite its increased effectiveness over its predecessors, YOLOv3 still lacks temporal information consideration, making it less suitable in dynamic environments [173].

**Figure 3:**
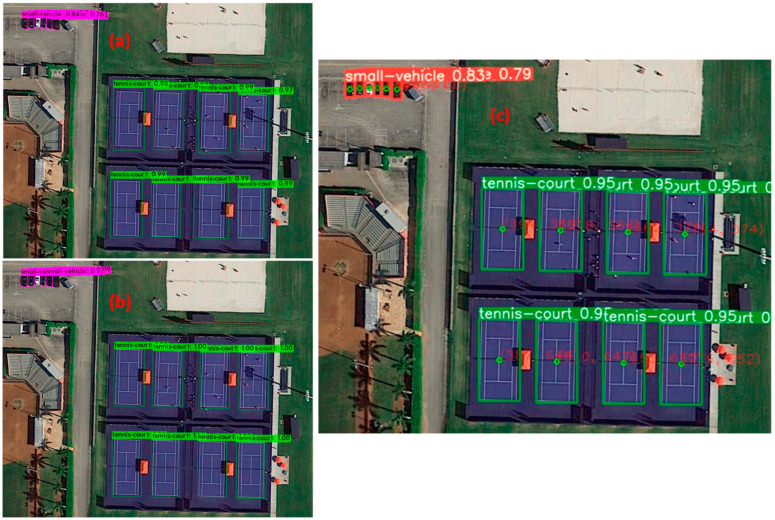
UAV target tracking: (**a**) YOLOv3, (**b**) YOLOv4, and (**c**) YOLOv5 [177].

The consecutive YOLOv4 is mainly characterized by three grids as follows: backbone (utilizes the CSPDarnet53 classifier), neck (a parameter assembling approach within lessen the information trajectory), and head (same as in YOLOv3). The evaluation target in YOLOv4 is improved compared with its predecessors via Generalized Intersection over Union (GIoU), which is used as a loss function [174]. As compared with YOLOv3, the backbone architecture in YOLOv4 enhances feature extraction with improved accuracy because of its progressive training with larger image sizes [175]. Forwardly, YOLOv5 represents a more streamlined model since it removes the CSPDarknet53 backbone, in addition to focusing on model size reduction [176]. As a visual interpretation, Figure 3 presents the graphics of the same objects detected by YOLOv3, YOLOv4, and YOLOv5 [177], where the boundary boxes become more precise with the evolution of each YOLO (i.e., by means of YOLOv5, the boundaries of each tennis court become tighter and more realistic with respect to the predecessors, higher degree of confidence of the objects’ classes, etc.). Moreover, YOLO-based target tracking algorithms kept evolving toward YOLOv6 [178], YOLOv7 [179], and YOLOv8 [180], aiming to have better object detection accuracy than each predecessor. The comparative assessment of YOLOv6, YOLOv7, and YOLOv8 is represented in Table 4.

#### 4.1.3. Payload Integration and Control

Depending on the UAV flight mission (i.e., the purpose of the flight), additional equipment (e.g., sensors, packages, etc.), referred to as payload [186], can be embedded with it in order to fulfil its mission’s objectives. In other terms, payload refers to any added weight or equipment to the UAV beyond its essential components for flight. With that being said, a payload can disturb a UAV’s proper flight, representing an external physical trigger for swinging and other forms of physical perturbations [187]. Therefore, payload control aims to operate and control the added weight, thus ensuring a UAV’s stable flight. For that purpose, suspended payload perturbation can be suppressed by means of a Nonlinear Disturbance Observer (NDO)-based neoteric anti-disturbance control strategy [188]. For the case of a cable-suspended payload, the full nonlinear dynamic behavior of a UAV is stabilized through a nonlinear control technique with a repetitive algorithm: reference points are tracked via back-stepping and integral sliding mode control, where the iterative algorithm shuts the error terms down to zero [189]. Similarly, for suspended-load systems, the UAV’s flight is stabilized through a nonlinear controller with the following working mechanism [190]: at each iteration, the UAV’s dynamic model undergoes an approximate linearization (i.e., relying on first order Taylor series expansion) at equilibrium; an H-infinity feedback controller is then designed for the resulting approximately linearized model. This results in fast and accurate tracking of the entire UAV system (incorporating the payload). The stability of the control scheme is justified with Lyapunov analysis [190]. Many other embedded payload solutions in UAVs exist [191]. Hence, it is easier to choose a control technique, where payload control is imperative for all tasks demanding high precision, adaptability, and real-time decision-making, expanding the possibilities for aerial applications.

A UAV control subsystem is diversified in nature, where its main aim is to ensure an effective flight, most importantly with autonomous operation [192]. Regardless of the UAV type (e.g., fixed wing [193], rotary wing [194], etc.) and its corresponding field of application (e.g., surveillance, packet delivery, etc.), its corresponding mission must successfully bypass obstacles and concisely follow the pre-planned path. AI-based navigation strategies excel in the adaptation to dynamic environments as compared to vision-based techniques. On the contrary, vision-based navigation techniques require less training than AI models. From another perspective, multi-fusion and bio-inspired path planning methods demand higher computational facilities than their relatives because of the fact that they involve data integration. As for the localization criteria, VO focuses on incremental changes, while RVL is based on local references and AVL relies on global coordinate systems. Considering target tracking, the reviewed models of the YOLO algorithm present real-time processing and detect objects based on the entire image context, but they still show less tracking ability in complex environments when compared with other specialized algorithms such as Kalman Filter [195], and Deep-SORT [196]. The payload deployment on the final destination cannot be generalized under defined methods or techniques since it is seen that such a criterion mainly depends on the geometry of the load, its weight, its installation/release mechanisms, and other application-specific constraints.

### 4.2. Computing

In relation to the previous control subsystem, the presented algorithms for a UAV’s navigation (whether vision or AI-based), as well as for path planning, obstacle avoidance, localization, target tracking, and payload deployment, need physical onboard systems for treatment. In other words, the execution of such algorithms is performed through computerized systems inside the UAV, serving as data processing, data logging, and all forms of diagnostics. Additionally, the raw data provided by the onboard sensors, and communication protocols with external parties, are also processed in the computing subsystem. In this context, a UAV’s computing subsystem encompasses two main sets including Single-Board Computers (SBCs) and System on Module (SoM).

(a) SBCs

As its name indicates, an SBC represents a complete computer that is built on a single circuit board, including a Central Processing Unit (CPU), Random Access Memory (RAM), storage, Input/Output (I/O) interfaces, and other features similar to a functional computer. Because of the fact that SBCs have smaller form factors, they are found to be compact and suitable for usage in space-restricted applications, such as in a UAV [197]. For UAV process management, the most common SBCs are Raspberry Pi, Odroid XU4, and NVIDIA Jetson (nano, TX2, Xavier).

*Raspberry Pi*

**Figure 4:**
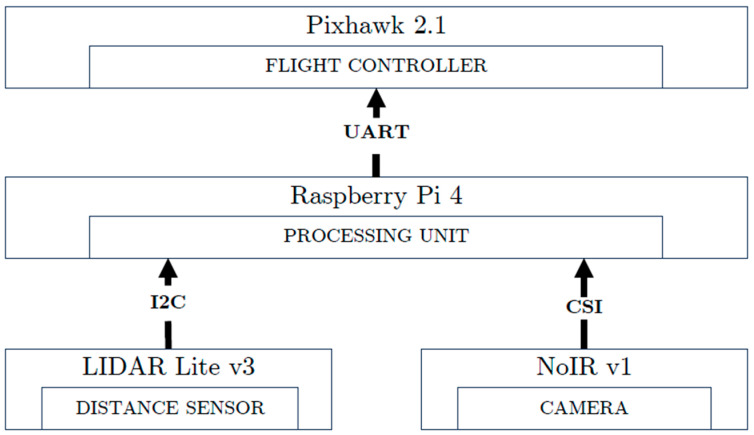
Overall block diagram representation of the control subsystem with field sensors [199].

Beginning with Raspberry Pi 4, it represents a new Raspberry Pi-based SBC that is increasingly involved in UAVs. This model is powered by a Broadcom BCM2711 quad-core ARM Cortex-A72 processor, which can be clocked at up to 1.5 GHz. It is available with up to 8 GB of Low Power Double Data Rate 4( LPDDR4) RAM, thus providing a stronger ability to deal with a UAV’s multitasking applications. Featuring a VideoCore VI GPU, supporting OpenGL ES 3.x, smoother graphics are rendered, hence better detecting objects and avoiding obstacles in UAV’s path planning. Aside from Universal Serial Bus (USB) and ethernet connectivity, Raspberry Pi 4 presents a built-in dual-band Wireless-Fidelity (Wi-Fi) of 2.4 GHz and 5 GHz, in addition to Bluetooth 5.0, hence presenting flexible communications with UAV remote parties. The storage capacity (e.g., used for video and images records) can be expanded by means of microSD cards, where its 40-pin General Purpose Input–Output (GPIO) facilitates connections to other computing platforms and sensors [198]. In the field of UAV applications, maneuvering was improved when an open source and MAVLink communication were encoded onto a Raspberry Pi 4 onboard SBC with a Pixhawk Cube 2.1 flight controller. This integration is shown in Figure 4 [199].

Figure 4 shows how Raspberry Pi 4 can flexibly make decisions concerning a UAV’s flight by dynamically adapting to different communication protocols with only a few subsystems (e.g., sensors). The data recorded with Raspberry Pi 4, clearly indicates the accuracy in UAV object detection [199]. For an object tracking-based UAV mission, when the Patch Color Group Feature (PCGF) framework was embedded on a Raspberry Pi 4, it resulted in 17 FPS offering a good execution speed with low PCGF computational complexities [200]. Older Raspberry Pi models, such as Raspberry Pi 2 B+, are effective in illustrating the relationship between time constraints of real-time systems and the analysis of temporary computational complexity [201], hence better managing failure possibilities in real-time processes.

*Odroid XU4*

**Figure 5:**
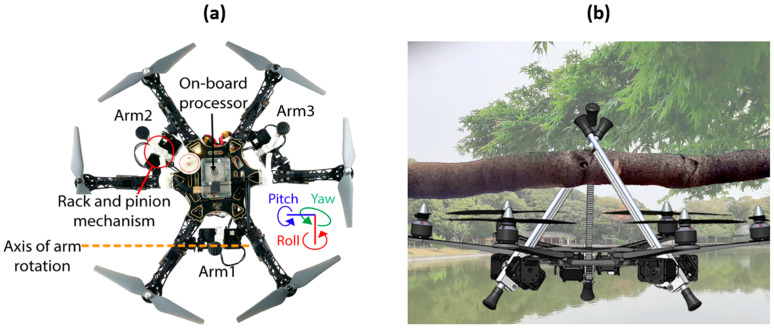
UAV (**a**) with Odroid XU4 as the on-board processor and (**b**) aerial docking [203].

As an alternative to Raspberry Pi as a UAV’s SBC, Odroid XU4 possesses a Samsung Exynos 5422 octa-core processor, consisting of four ARM Cortex-A15 cores, clocked at 2 GHz. The remaining cores consist of ARM Cortex-A7 clocked at 1.4 GHz. This core’s combination provides a trade-off between performance and energy efficiency. This SBC comes loaded with 2 GB of LPDDR3 RAM, which is sufficient for multitasking and general UAV processing applications. Graphics are featured via a Mali-T628 MP6 GPU, thus proving an ability to withstand UAV-based decent graphics (i.e., taken through cameras). In addition to USB and Ethernet communication modules, Odroid XU4 supports Wi-Fi communication via an external USB adapter. Instead of a 40-pin GPIO, as in the case of Raspberry Pi, Odroid XU4 presents a 30-pin GPIO header, hence reflecting lower accommodation with external devices and sensors. One major drawback of this SBC compared with Raspberry Pi is that it produces significant heat under heavy computational loads, hence implying the need for a cooling utility (e.g., fan, heat sink, etc.) and posing more challenges in front of space limitations [202]. The on-board processor shown in Figure 5a represents an Odroid XU4 deployed to provide the motor control operations [203].

For the Odroid XU4 used in the UAV in Figure 5, its docking mechanism (i.e., over a tree branch, as in Figure 5b) is designed by means of a three-arm manipulator, thus allowing for resting periods (for whatever reasons) during its flight. From a different perspective, for visual-based detection as well as tracking cooperative UAVs, Odroid XU4 can embed an algorithmic architecture based on YOLOv2. Computational time and false alarms can hence be reduced through navigation data exploitation from tracker-target UAVs. Over 90% of target line-of-sight were correctly detected and accurately estimated [204].

*NVIDIA Jetson*

**Figure 6:**
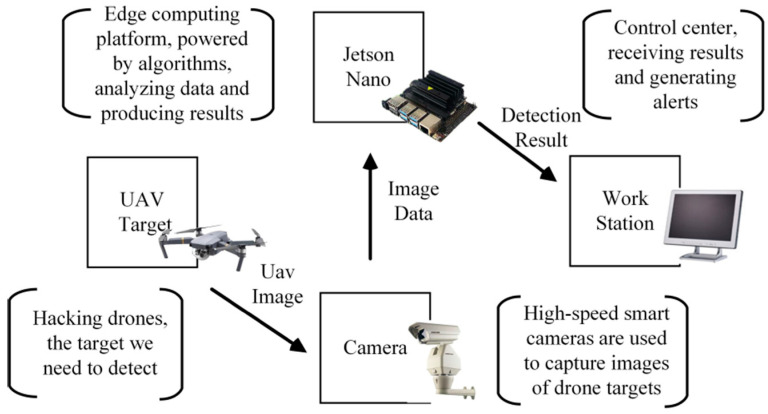
Proposed UAV object detection system based on NVIDIA Jetson Nano [207].

NVIDIA Jetson is a series of SBCs designed specifically for embedded applications, presenting a leading AI-compatible platform in a compact and energy efficient form factor. An NVIDIA Jetson SBC includes a dedicated GPU, optimized for parallel computing, sensor fusion, computer vision, etc. As a sub-model, Jetson TX2 represents a supercomputer-on-module with up to 8 GB of LPDDR4 RAM and a dual-core NVIDIA Denver 2 CPU with quad-core ARM Cortex-A57. The NVIDIA Pascal graphics processor with 256 cores represents its GPU, thus allowing for higher visual resolutions [205]. In a drone-based pedestrian tracking process [206], Jetson TX2 enables real-time tracking while effectively addressing challenges relating to computing power limitations. Additionally, it achieves the real-time processing of DL-based object tracking tasks. This is mainly performed with assistance from both CPU as well as GPU integrated within Jetson TX2, showing high efficiency in target tracking based on 4K video streams captured by the UAV at an elevation of 50 m. Another model of NVIDIA Jetson SBCs, Jetson Nano, is found to be capable of embedding an improved version of YOLOv4, the Fast-YOLOv4 [207]. Figure 6 elaborates the conjunction of Jetson Nano with other components of the object detection system.

The main role of the Jetson Nano in Figure 6 (i.e., 128-core CUDA Maxwell GPU, 4 GB LPDDR4 RAM, quad-core ARM A57 1.43 GHz) is the real-time analysis of image data, in addition to sending abnormal results to the workstation through Wi-Fi. The resulting mAP reached 90.62% with a 54 FPS [207]. Table 5 provides a comparative assessment of the different reviewed SBCs used in UAV processes.

(b) SoM

A SoM reflects an integrated computing platform encompassing the essential component of a computer (e.g., CPU, RAM, storage, etc.) onto a single module, simplifying the build of larger embedded systems. While both SBCs and SoM serve for similar purposes in embedded systems (e.g., flight control of a UAV), they differ in terms of the form factor and design architecture. On one hand, SBCs come packaged with all essential components, which makes them standalone computing systems, thus neglecting any need for additional hardware. A SoM, on the other hand, cannot directly connect to peripherals because it typically does not have I/O sockets [208]. For UAV control, different SoMs can be used such as NXP I.MX8M [209], Rockchip RK3399 [210], Qualcomm Snapdragon [211], and STM32 [212]. Regarding the work in reference [213], NXP I.MX8M provides a custom AI video-processing-focused hardware platform for the deployment of DL models. By integrating a Neural Co-Processing Unit (NCU), it enables the process of 2.3 tera operations per second, hence considerably reducing the processing time for the implemented DL models used in smart cities’ smart-camera-systems. Similarly, in [214], Rockchip RK3399 serves as the SoM for video data (recorded in real time by a UAV) processing, providing more speed and prohibiting delays in video transmission in 5G networks. For the purpose of UAV safety landing in a GPS-denied environment, the work in [215] utilized a “remote-marker-based” tracking algorithm that is implemented on a Qualcomm Snapdragon SoM. The developed CNN “LightDenseYolo” algorithm, by means of an embedded Qualcomm Adreno 540 GPU, extracts features from an input image. This extraction can be used to predict a marker’s location by a visible light camera sensor installed on the UAV. The suggested methodology outperforms state-of-art UAV object trackers [215].

Concerning a different approach than in the previously mentioned works, the work in [216] emphasized monitoring a mobile network (i.e., performing testing and measurements) by means of a UAV through the Galilelo satellite network. The proposed software runs on a Qualcomm Snapdragon SoM (embedded in a Xiaomi mi 10 lite), enabling effective and accurate remote extraction of mobile network data (e.g., signals levels, data logging, information visualization, etc.). The authors of the reference [217] take a different perspective, based on an improved algorithm involving greater immunity versus cyber attacks in the Internet of Drones (IoD) on a Qualcomm Snapdragon SoM. Their results indicate a stubborn protocol with good resistance in front of security attacks of a swarm of drones used for smart city real-time data collection. Alternatively, in reference [218], the aim is to rescue injured humans swiftly by means of mission choice-driven UAV swarm cooperation. After the transfer of a suspected target’s location to a self-organizing network, a bio-radar UAV rechecks for any survivors via a respiratory characteristic-based algorithm that is processed by a STM32 SoM. The data packets sent by the STM32 eventually allow the emergency supplied drop-off by means of a nearby emergency UAV according to the location/vital signs of the target, showing good success and accuracy. Specifically, the STM-based SoM finds various applications in UAV processes. For instance, aside from injured human targeting and help, STM32 succeeds in the analysis of a quadcopter’s stability, first, by reading data of angular velocity and acceleration. Then, by supplying Pulse Width Modulation (PWM) signals to an Electronic Speed Control (ESC), the brushless DC motors are correspondingly regulated, providing more controllability and a reduction in the response time of the UAV [219]. These SoM also find use in human–UAV interactions based on machine learning in wearable gloves [220]. With one STM32, five flex sensors, and one Inertial Measurement Unit (IMU) sensor installed in each glove, gestures are recognized and equivalently translated in a control command for the UAV.

The variety of SoMs that can be employed in a UAV process, as can be concluded from the small survey of the literature presented above, makes their selectivity criterion challenging, especially since each comes with specific pros/cons. Therefore, for an optimal SoM selection, a standard assessment must be conducted in which standard factors, such as programming complexities, communication protocols, form factors, and, most importantly, the UAV’s mission objectives, must be taken into consideration. This required assessment is presented in Table 6. It is worth saying that after analyzing Table 6, it can be concluded that some SoMs may excel in demanding less computational power, while others prioritize power efficiency. For some UAV applications, mainly related to network monitoring, and integration capabilities (i.e., ability to seamlessly communicate with remote parties, sensors, etc.) Qualcomm Snapdragon would be prioritized the most.

The assessment presented in Table 6 adds a straightforward approach to an optimal selection of a SoM in a UAV process. For instance, when the selection is mainly based on the UAV’s flight purpose, on one hand, for aerial surveillance, the Qualcomm Snapdragon can be the best candidate because of its powerful GPU performance (i.e., high-definition real-time video processing). Additionally, its support for 5G connectivity ensures fast data transmission, as required for real-time surveillance. Other UAV missions can involve package delivery, on the other hand. Aside from parcel delivery, emergency kits, medical supplies, and packages can, for example, the payload consists of electrical sensors to be deployed on the overhead electrical transmission lines. Accordingly, such applications would require accurate navigation and good management of the payload’s weight. For such particular applications, Rockchip RK3399 is found to be optimal, according to Table 6. This is mainly because it offers a trade-off between high performance and economic power consumption, hence achieving autonomous navigation while optimizing battery life. For longer missions (e.g., crops assessment and agriculture) that require high real-time processing capabilities, STM32 is found to be the best candidate among the others because its very low power consumption extends the UAV flight’s time, along with its integrated ability for high data collection.

### 4.3. Communication

Between the UAV and external entities (e.g., Ground Control System (GCS)), a bidirectional data exchange is enabled by means of the communication subsystem. For applications involving a swarm of drones, it also enables mutual communication and data exchange between each UAV, transmitting control commands, captured images, and other data. It might be the case that the computing subsystem processes the data from sensors through the communication subsystem; however, the focus in this paper took into consideration only remote communication with external parties. Based on this approach, the communication modules/protocols were found to include Long Range (LoRa), Wi-Fi, Bluetooth Low Energy (BLE), and Long-Term Evolution for Machine-Type Communication (LTE-M).

(a) LoRa

LoRa refers to long-range low-power wireless communication technology allowing for telemetry between a UAV and an external party [221]. Its physical layer employs a private Chirp Spread Spectrum (CSS), with LoRaWAN as its MAC layer [222]. Within the framework of UAVs, the identifier of a UAV is transmitted with real-time status tracking through LoRa [223], as well as other data concerning cases of environmental disasters [224]. From a different perspective, LoRa is utilized along with UAV-embedded IoT devices to track merchandise and increase their location accuracy in [225], whereas in [226], LoRa was employed to link a UAV with air pollution monitoring stations. Although LoRa presents the capability to transmit for up to 15 Km in rural areas [227], over unlicensed bands at frequencies ranging from 433 MHz to 923 MHz, it suffers from a low data rate, ranging between 0.3 and 5.5 kilobytes per second (kbps) [228]. This communication technology can be enabled inside a UAV through different modules, for example, SX1278 [229] supporting the frequency range from 137 MHz to 525 MHz. Alternatively, RN2483 [230] can also enable LoRa inside a UAV with a data rate of 300 kbps, similar to HOPERF RFM95W-868S2 [231], which works on a frequency of 868 MHz.

(b) Wi-Fi

In lieu of LoRa, Wi-Fi enables remote control in UAVs, in addition to real-time data transmission, telemetry with GCS, and communication with other UAVs [232]. Wi-Fi communication in UAVs usually follows the Open System Interconnection (OSI) model, consisting of seven layers [233]. Within the framework of UAVs, the wireless systems’ range (i.e., coverage zone) is extended through an intel Galileo board installed in a UAV, where Wi-Fi is configured in ad hoc mode [234]. A regional inspection and monitoring system based on Wi-Fi is established in reference [235], in which the signal strength model based on the Wi-Fi directional gain antenna is introduced. The leader–follower trajectory scheme in [236], on the other hand, implements a Robot Operating System (ROS) based communication system for a swarm of UAVs via Wi-Fi and MavLink serial forwarding. Tests result in fast responses between workstation–leader and leader–follower communication (0.2 S and 0.42 S, respectively). Unlike LoRa, which is featured by a coverage zone in kilometers, Wi-Fi technology has a limited range (maximum of 300 m outdoors). From another perspective, Wi-Fi presents an elevated throughput data rate, which varies according to each protocol (e.g., 802.11ax, 802.11ac wave2, 802.11ac wave1, 802.11n, 802.11g, 802.11a, 802.11b, [237]). Different modules exist that enable Wi-Fi communication on a UAV, of which ESP8266 [238] enables the SBC/SoM to connect to 2.4 GHz via the 802.11bgn. A better alternative to ESP8266 is ESP32 [239], which presents a Bluetooth connectivity, also featuring a dual-core processor allowing for better multitasking. CC3000 [240] can alternatively add Wi-Fi functionality to a UAV, supporting the 802.11b/g protocol, but, on the other hand, it does not support the AP mode.

(c) BLE

Representing an enhanced version of Bluetooth, BLE is designed for applications that require short range demands with low power, having the same bit rate, frequency range (i.e., from 2.4 GHz to 2.48 GHz), and range of traditional Bluetooth [241]. In combination with the Received Signal Strength Indicator (RSSI) technique, the BLE-RSSI combined method presented a lack of precision in UAV positioning, which was overcome in [242]. With regard to UAV navigation in GNSS-denied environments, an indoor positioning system for UAVs is set up through BLE beacons by analyzing the RSSI, where the final position estimation is acquired by trilateration [243]. In the catapult launcher of small UAVs in [244], BLE consisted of a positioning feedback system, providing a continuous connection between the user and the control panel. BLE can be added to a UAV through the nRF54H20 System-on-Chip (SoC) which presents multiple ARM Cortex-M33 processors with a 1 MB of RAM, providing a long range with a 10 dBm of transmission power [245]. The nRF54LI5 SoC from the same series, on the other hand, comes with a single 128 MHz Arm Cortex-M33 processor and 256 KB of RAM, with 8 dBm of transmission power [246]. Different from the nRF54-based series, CC2650 [247] represents another medium with which BLE communication can be enabled in a UAV, and it comes equipped with a 20 kB of RAM and up to 31 GPIO ports.

(d) LTE-M

A low-power, wide-area, cellular-technology-based communication supports machine-to-machine interaction as well as IoT. LTE-M presents a high data rate and an increased bandwidth and can be easily integrated with existing cellular networks [248]. By means of LTE-M, the range of UAV operations can be extended by the supported Beyond Visual Line of Sight (BVLOS) [249]. In [250], the performance of LTE-M was investigated with a UAV for a distance of 450 feet, where 5G communication was found to have better signal strength. To equip UAVs with LTE-M communication, Quectel BG95-M3 LGA [251], on the one hand, presents a good solution featured by an ultra-low power consumption as well as multiple frequency band support. Telit ME310G1-WW [252], on the other hand, adopts another low-power operation by adapting a specific wakeup–transmit–sleep mechanism, also supporting various LTE bands and GNSS technologies.

According to the different surveyed communication technologies that can be employed in a UAV, the choice for a specific module based on communication theory/protocol can be challenging. It is true that all methods share the wireless connectivity criterion, but still, many other factors must be taken into consideration when embedding a communication module in a UAV. Firstly, the range of communication (i.e., coverage zone) reflects a great impact factor, and it can be noticed between LoRa and BLE. Environmental constraints (e.g., signal degradation, deterioration, and complete loss) are another key factor for the choice of the surveyed modules. How to program each module is another topic, since the programming requirement is another decisive factor. LoRa-based modules can cover wide zones with a high bitrate, but their supported frequencies may not intersect with those received in the ground/remote station. LTE-M based modules, on the other hand, possess the greatest bitrate (i.e., optimum for high data exchange) but are heavily dependent on network coverage. Table 7 provides a standardized assessment of each physical module in each of the four main communication sets surveyed (i.e., as much as applicable since some differ intrinsically).

### 4.4. Sensory

**Figure 7:**
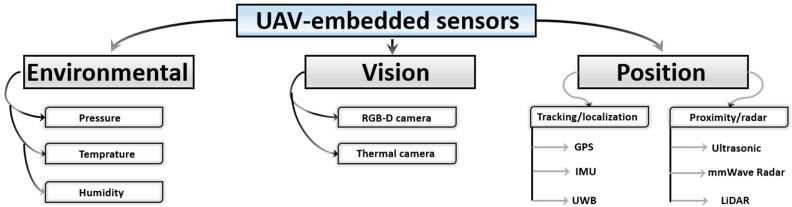
Overview of the different reviewed UAV sensors.

Sensors in UAVs represent the data source for each of the previous main subsets. In other terms, the physical data acquired from different sensors are computed in the decision-making control subsystem and communicated (i.e., when necessary) with other internal/external parties. The level of autonomy in a UAV’s application (i.e., fully autonomous [253], semi-autonomous [254], or human-controlled [255]) majorly depends on the type/number of sensors. For instance, autonomous UAVs are found to be capable of executing missions without human intervention, which is related to their installed wide array of sensors, thus allowing a better understanding of the surrounding environment. Additionally, a popular case is sensor fusion in autonomous UAVs, where combined data improve perception and self-decision-making [256]. Manually controlled UAVs, on the other hand, have minimal sensor payloads since they follow a human-determined path where their embedded sensors are mainly for emergency cases (i.e., electric power dropouts, etc.). Here, the surveyed sensors that can be installed onboard UAVs are mainly classified as either environmental, vision, or position sensors, as shown in Figure 7.

*Environmental sensors*

(a) Pressure sensors

A UAV’s flight control and navigation are affected by the data provided by the environmental sensors, such as temperature, humidity, and pressure. For instance, safe flight levels are maintained via the altitude information provided by the pressure sensors. Altitude measurements are generally challenging to obtain, especially because of vibrations and fast-changing environmental conditions. In [257], these challenges were first addressed by focusing on the UAV’s vertical movement measurements. The altitude was finally determined via the data obtained from the barometric pressure sensor MS5611, fused with other data from an ultrasonic sensor. MS5611 is a high-resolution barometric pressure sensor (24-bit Analog to Digital Conversion (ADC)), with a pressure accuracy of ±1.5 mbar and an operating range from 10 to 1200 mbar [258]. Another digital pressure sensor, BMP388, can also be UAV-embedded with a less sensitive operation range between 300 and 1250 mbar and has a typical relative accuracy of ±0.08 mbar [259]. This BMP388 finds application in UAV automative radar for earth/land monitoring processes [260].

(b) Temperature sensors

Apart from pressure sensors, temperature sensors help in the monitoring processes of ambient conditions. Thus, they ensure an optimal performance of onboard electronics as well as propulsion systems by declaring fault signals in the case of over-/underheating conditions. On the one hand, DHT11 (combined temperature and humidity sensor) collects corresponding environmental data from a specific location, which is dedicated to being sent to a webserver for remote monitoring [261]. Other temperature sensors, such as the one in reference [262], are used for calibrating the grey in the photos taken from thermal cameras. In [263], on the other hand, temperature sensors were found to be (in addition to other sensors) effective in soil monitoring and the proper management of crops.

(c) Humidity sensors

Similar to the work in [261], the combined temperature–humidity DHT11 sensor was used in [264,265] to create an efficient IoT-based weather station. As an alternative to DHT11, SHT75 [266], representing a digital pin-type humidity sensor with ±1.8% typical relative humidity accuracy, was used in [267] to measure air humidity for the purpose of constructing an intelligent weather station. From a different point of view, SHT40 in [268] participated in the construction of pressure–temperature–humidity probes for distributed atmospheric monitoring via a UAV. Lastly, a relative humidity sensor was employed in [269] as a sub-component from a massive project aimed at monitoring the temperature and humidity in Antarctica.

It can be generally deduced that humidity sensors help to ensure a stable UAV flight by preventing condensation-related problems. The synergistic operation of the three environmental sensors enhances a UAV’s situational awareness, hence allowing a dynamic accommodability to changing environmental conditions. Additionally, environmental sensors can have external duties that are not related to UAV navigation and flight safety, mainly as contributions to effective weather stations, as stated in the survey of the literature stated above.

*Vision sensors*

Different from environmental sensors, vision-based sensors provide a visual perception of a UAV’s surrounding environment by generating an image of the captured scene. The processed image is then forwarded to its relevant computing subsystem in order to make a decision based on it in the control subsystem. For example, the raw version of the obtained image can be processed through any of the SBCs (e.g., Odroid XU4, NVIDIA Jetson), where, by means of the YOLOvx navigation control algorithm, targets can be efficiently tracked while obstacles avoided. Two of the most used vision-based sensors in UAVs are RGB-D and thermal cameras.

(a) RGB-D Cameras

An RGB-D camera provides a perception (i.e., closely related to human visual perception) of a UAV’s surroundings in the form of RedGreenBlue (RGB) images. As previously mentioned, targets can be tracked, and the UAV’s localization can be acknowledged upon the processing of RGB images to computer vision algorithms through SBCs/SoM. Spatial distances of targets can also be calculated via the RGB images’ depth information, thus providing better collision avoidance strategies. Overall, such a camera’s quality is assessed from its frame rate, aperture, and shutter type [270]. One type of RGB-D camera is the Intel Realsense D435 [271], which was found to be ideal for fast moving applications in low light and a wide field of view. It has an RGB frame rate of 30 FPS and an RGB frame resolution of 1920 x 1080. Intel Realsense D435 was used in reference [272] to collect depth measurements in order to identify the parameters of the depth camera noise model, thus enabling more accurate SLAM algorithm execution. RGB-D cameras can also be involved in 3D mapping to describe a UAV’s surroundings after SLAM fusion with a UAV [273].

(b) Thermal Cameras

**Figure 8:**
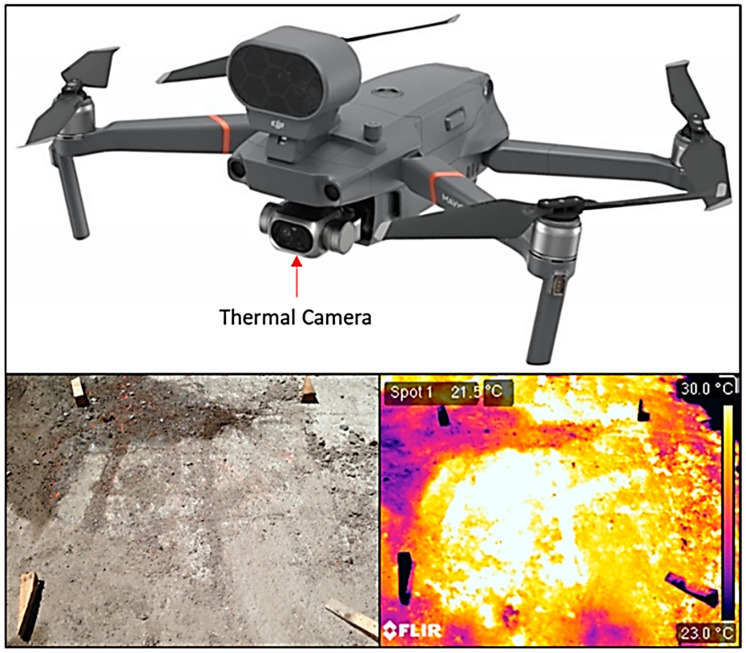
Sample thermography data obtained from a UAV-embedded thermal camera [275].

A thermal camera is an equipment that captures and detects Infrared Radiation (IR) emitted by surrounding objects. This type of camera allows for imaging based on heat, which can be used for aerial surveillance, environment monitoring, and search–rescue processes [274]. Thermal cameras find applications in different UAV processes, such as the bridge infrastructure assessment shown in Figure 8.

As shown in Figure 8, a bridge’s infrastructure non-destructive testing by means of a UAV saves time and gives more accurate and faster results than visual inspection. The actual captured image of the bridge (Figure 8, bottom left) is reflected in an equivalent thermal image (Figure 8, bottom right), revealing the cohesion between different parts of the bridge.

*Position sensors*

UAV movements are detected via position sensors, where the latter provide spatial information with respect to a defined reference. Such sensors are generally used in UAVs to pinpoint their precise location in order to later share it with the user/control operator (for cases of semi-autonomous and human-controlled UAVs) or to compute it in the navigation control (for cases of autonomous UAVs). Therefore, position sensors determine the orientation of a UAV in addition to providing odometric information about it [276]. In this paper, position sensors are mainly classified according to their purpose of usage/outcome, characterized as either tracking/localization or proximity/radar.

(a) Tracking/localization

**Figure 9:**
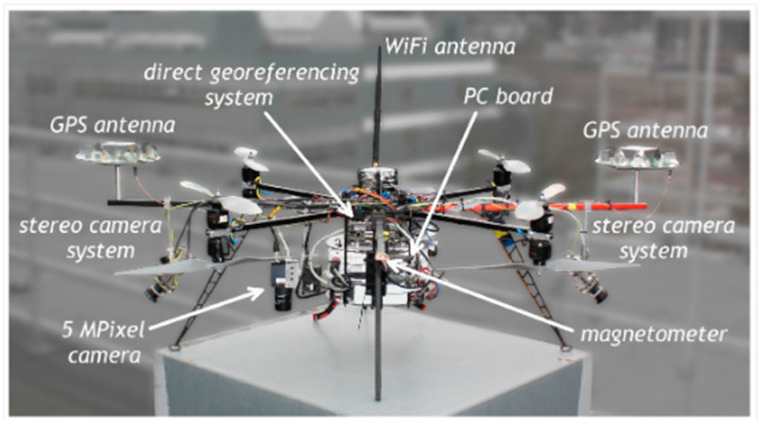
UAV with added UWB modules and other sensors [283].

The localization-based navigation strategy as well as the target tracking scheme under the control section obtain their inputs from sensors such as GPS, IMU, and Ultra-Wide Band (UWB) [277]. On one hand, GPS sensors provide accurate time–space information for UAVs. Specifically, the Real-Time Kinematic (RTK) GPS technique provides positioning updates with high frequency and is thus able to withstand a UAV’s swift velocities and high maneuverability [278]. For instance, a high-precision RTK GPS was used in [279] for the purpose of determining the locations of a ground control target. In addition to localization, GPS modules embedded in UAVs provide temporal information, which can also be used for stabilization. The u-blox NEO-M8N [280] representing a low-power compact form GPS receiver, was used in [281] to solve the problem of wind gusts with their adverse effects on UAV steering while inspecting transmission lines. On the other hand, IMUs are most probably found in UAVs in the form of accelerometers, gyroscopes, and magnetometers [282]. Along with GPS modules, IMUs form INS, which is responsible for localizing, stabilizing, and tracking a UAV. In the work [283], with its UAV shown in Figure 9, the corresponding IMU (i.e., GPS antenna, magnetometer) had the function of attitude determination, localization, and navigation of a UAV.

In addition to the GPS and IMU modules, UWB sensors utilize pulses of short-range radio frequency to determine the location of nearby objects. The location of a target is known when the UWB receiver receives (according to a specific frequency spectrum) the corresponding pulses sent by the UWB transmitter [284]. For positioning, UWB utilizes either the time difference of arrival approach [285] or the two-way ranging method [286]. DWM1000 [287] is a commonly used UWB transceiver module, supporting four radio frequencies from 3.5 GHz to 6.5 GHz, with a data rate up to 6.8 Mbps. In the work [288], the DWM1000 orchestrated ranging measurements between a UAV and fixed anchor nodes, eventually localizing the UAV precisely. This suggests how UWB can help in positioning UAVs in GPS-challenging environments.

(b) Proximity/radar

In short, proximity sensors provide spatial information with respect to objects existing nearby in their field (i.e., detecting objects in short ranges via IR and other ultrasonic technologies). Radar sensors, on the other hand, use radio waves to detect objects located at greater distances than those in the previous case. Concerning ultrasonic proximity sensors, HC-SR04 [289] provides from 2 cm to 400 cm of non-contact distance measurements with an accuracy of ±3 mm. Such a sensor was used in reference [290] to detect nearby objects of an indoor UAV. As an alternative, MaxSonar MB1222 [291], with a higher range in object-detection than the previously mentioned sensor (i.e., 20 cm to 765 cm), finds application in indoor localization of micro-UAV, with accurate performance [292]. From a different perspective, mmWave radar sensors provide accurate target range and velocity information, surpassing the other alternatives under extreme weather conditions [293]. One example is IWR6843, which provides a range of detection of 180 m and an ±120 elevation field of view [294]. At the final destination, and although LiDAR mainly aims at generating 3D maps of the environment (via analysis of the reflect light from emitted laser pulses), it can as well be utilized for UAV positioning (e.g., providing precise measurements of a UAV’s altitude above ground level [295]). For instance, Livox-avia [296] represents a LiDAR sensor with a 450 m maximum detection range. This sensor was used in reference [297], in which a UAV’s real-time positions were identified based on the utilization of it with RGB-D cameras (i.e., visual-LiDAR fusion).

Amongst the different reviewed UAV sensor types, environmental sensors (i.e., measuring temperature, humidity, and pressure) possess an additional role (to data sharing) in ensuring flight safety. For example, a UAV flight can be urgently terminated or have its span-reduced under excessive rain or humidity. On the other hand, vision-based sensors serve primarily for data extraction. The captured visual information aids in navigation tasks by detecting objects and avoiding collision with them. Regarding a UAV’s position sensors, they directly enable accurate control via the information about the UAV’s position, velocity, and orientation. Regardless of the sensor type, the more embedded sensors are within a UAV, the better its accuracy in maintaining a safe autonomous flight.

### 4.5. Power

As can be seen in Figure 1, the power subsystem is the most critical amongst all the others, representing the operating fuel for every SBCs, SoM, communication, and each sensory module. A UAV’s flight pace and duration are mainly dictated by the offered energy level from the power subsystem. In order to successfully overcome limited flight endurance, the power system must be carefully chosen, mainly based on the UAV’s purpose of flight. For example, a search-and-rescue UAV requires more power than the one used to assess public infrastructure since the first often interacts with spontaneous incidences of high risks. Generally, the power source of a UAV must be chosen according to its durability, it must have a good energy to weight ratio and create as minimum noise and vibrations as possible, and it must be easily replaced. Conventionally, UAVs are powered with batteries, battery hybrids with proton membrane fuel cell, gasoline engines, hydrogen fuel cells hybrid with Lithium-Ion batteries (Li-I), and solar power.

**Figure 10:**
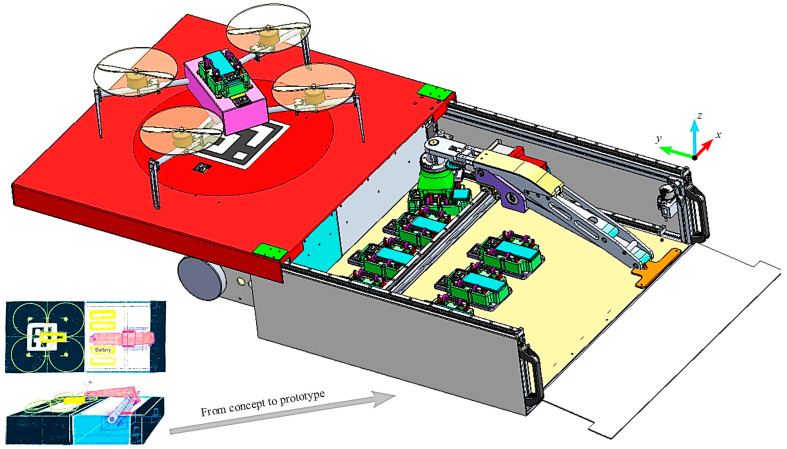
Droneport schematic representation [299].

Beginning with the work in [298], a 14 kg UAV had a power of 960 W supplied from a hybrid system, which was composed of a proton-exchange membrane fuel cell (as the main supply) and a Li-I (as a backup). This power-source mixture took advantage of the fuel cells fast refueling and ability to supply power for long distance ranges, in addition to the Li-I high acceleration and fast response to load variations. Such a power scheme, however, may present concerns regarding its longevity under frequent charging/discharging of the Li-I. An alternative way to overcome the UAV’s battery capacity issue and the need for its frequent charging is Droneport [299]. It consists of a landing platform, over which the UAV can have its battery exchanged and charged outside of its frame. As an alternative to hybridization with other-than-battery power sources, Droneport represents a complete electromechanical system, as shown in Figure 10, enriching the capabilities of UAVs with existing batteries power sources.

Unlike the study in [298], the work in [300] suggested a hybridization between gasoline and electric motors: two gasoline motors supplied the majority of the power needed for the lift force, with four electric motors utilized to stabilize the drone. Since the propellers consume the majority of the power within a UAV, the suggested gasoline–electric hybrid method prolongs a UAV’s flight time. Apart from gasoline, electric, and hybrid power sources, UAVs can be solar powered. In reference [301], a 2 m wingspan UAV was powered through a combination of solar PhotoVoltaic (PV)-based power and a battery. Under fair weather conditions, the solar–battery hybrid system saved up to 22.5% of the battery stored capacity.

Solar-based power supplies for UAVs are generally inconsistent since they mainly depend on solar irradiance, which can be very fluctuant. Even during sunny days, cloud-induced Partial Shading Conditions (PSCs) [302] impose great challenges in extracting the maximum available power from PV modules. Gasoline-based power strategies, on the other hand, induce fire hazards and higher maintenance requirements, regardless of the extended flight capabilities. Modern research in Li-I optimization would constitute an optimum alternative for traditional as well as hybrid UAV power sources, concerning weight restrictions, explosiveness, extended Depth of Discharge (DoD), and other factors [303].

## 5. Discussion

**Figure 11:**
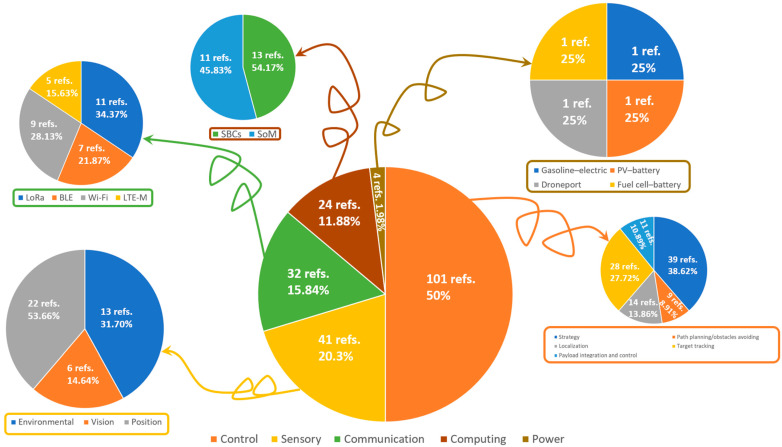
Statistics of the current review.

This complete review consisted concretely of 202 total references (since some references are only exemplary/explanatory) of which there were 101 references (50%) for the control subsystem, 41 references (20.3%) for sensory, 32 references (15.84%) for the communication subsystem, 24 references (11.88%) for the computing subsystem, and 4 references (1.98%) for the power subsystem. Under the control subsystem, there were 39 references for strategy techniques (38.62% of the total references, particularly within this set), 9 references (8.91%) for path planning/obstacles avoidance, 14 references (13.86%) for localization schemes, 28 references (27.72%) for target tracking, and 11 references (10.89%) for payload integration and control. Concerning the computing subsystem, 11 references (45.83% of the total, particularly within this set) were dedicated to SBCs, whereas 13 references (54.17%) were for SoM. For the communication subsystem, 11 references (34.37% of total references, particularly within this set) were for LoRa, 9 references (28.13%) for Wi-Fi, 7 references (21.87%) for BLE, and 5 references (15.63%) for LTE-M. In the sensory subsystem, 13 references (31.70% of total references, particularly within this set) were for environmental sensors, 6 references (14.64%) for vision sensors, and 22 references (53.66%) for position sensors. Lastly, four different power sources were classified in the final power subsystem, with each referring to a single reference (25% of total references, particularly within this set). This information is shown in Figure 11.

Most of the reviewed references refer to different control strategies, since such criteria are directly linked to overall flight stability and success. The employed algorithms for navigation, target tracking, localization, and obstacle avoidance represent the backbone that output modules (i.e., actuators) rely on, in relation with the data acquired through the sensors. Beginning with the vision-based navigation strategies, they pose a challenge on the computing subsystem, specifically concerning the GPU, in order to fully extract the graphical potential behind such strategies (i.e., high-resolution images with precise BRF), an SBC with high GPU capability (e.g., NVIDIA Jetson series) must be employed. In addition to financial burdens, the processing of image-based data incorporates extra programming complexities that are also naturally accumulated by AI-based techniques. The good part of the latter is that they allow for more autonomous UAV processes.

In the context of target tracking, the YOLO algorithm with its relative successors (i.e., up to YOLOv8), have shown popularity in adaptation for computing an object’s correlative and class probabilities. With every new version of YOLO comes major improvements in terms of knowledge accuracy with respect to its predecessors. For the part of the computing subsystem, the Qualcomm Snapdragon showed superiority with respect to all other SoM/SBCs, mainly regarding its RAM size, integrated GPU, built-in connectivity modules, and ability to be programmed in different languages. From another perspective, according to the data presented in Table 7, LoRa technology is the optimum selection for UAV flights requiring long range of communication (up to 20 km outdoors), with the highest data rate (up to 300 kbps). However, a trade-off must be made between its RAM size and transmission power to better fit an application (i.e., the 32 kB of RN2483 imposes a loss of 2 dBm in the transmission power). The golden rule concerning the sensory part corresponds to the following: the more sensors installed in a UAV, the better its environmental/internal awareness, and the more burden is then created on the level of the computing subsystem. Some sensors, such as temperature sensors, are unavoidable since their output data is highly influential on flight safety and stability. Sensors can also be fused together (i.e., sensor fusion) to provide better decision-making in the control subsystem, as a result of the data-dense outputs. Regardless of the different available power sources for UAVs, as surveyed in this paper, classic batteries are still the best form. For instance, Droneport is an innovative idea, but it lacks experimental validation under severe operating conditions, and it serves only in recharging/replacing a UAV’s existing battery. With the stated unreliability of PV supplies and dangers of gasoline motors, the optimum UAV power supply is an optimized Li-I battery [304]. In comparison with other UAV reviews, the contributions of this paper are shown in Table 8.

In addition to the compared literature presented in Table 8, more narrow reviews exist in the field, but they are denser in the sense of the exploited UAV aspects. For example, the dedicated review in reference [311] focuses mainly on the control of hybrid and convertible UAVs. With a technical modus operandi, emphasizing around vertical takeoff and landing (VTOL) UAVs, the presented review investigates commonalities and differences in guidance, modeling, and control allocation for each type (e.g., Tail-sitter, Tiltrotor, etc.) of hybrid VTOL UAVs. Unlike the methodology presented in this work concerning the control assessment, the work in [311] outlined four main components of a successful flight control system for VTOL UAVs, depicted by a physical model, generation of reference trajectories, flight controllers, and allocation of actuator control. Additionally, the work in reference [312] outlines a UAV-type addressed review, with a core focus on flight modeling techniques, along with flight control strategies. The review began by assessing the flight’s success for different miniature UAVs based on the control techniques. Moreover, further analyses were conducted based on the mechanical structure of the UAV and its corresponding control feature (e.g., tail-sitters with hovering control). As a major observation, the work conducted in [311,312] sheds light on the differences between their control sets and the one held in this paper. Accordingly, it can be said that despite the various contributions made by this paper, it still lacks many criteria in the domain of UAVs. The popularity of this trend, and its daily considerations and improvements, makes it impractical to grasp the required avionics information in one single paper. For this reason, and in order to remain within this paper’s maximum limits (in word counts as well in page numbers), this study did not survey any related manuscripts about the UAV mission field of application (i.e., no thorough investigations about surveillance, crop assessment, delivery, and other UAV’s missions). In addition, when reviewing the different aspects in this paper, it can be noted that a discussion was not provided on UAV type. Additionally, although this paper presents a wide variety of data processors (i.e., SBCs, SoM), no microcontrollers (e.g., Arduino, PIC, etc.) were involved in the analysis since not all missions require heavy computational facilities. For the sensors, this paper did not present any single mechanical or electrical sensor, which are heavily required and employed in UAVs; instead, the focus was merely on environmental, vision, and position sensors. Again, this was mainly because of the paper’s length restrictions and to maintain a readable review format.

## 6. Future Work

**Figure 12:**
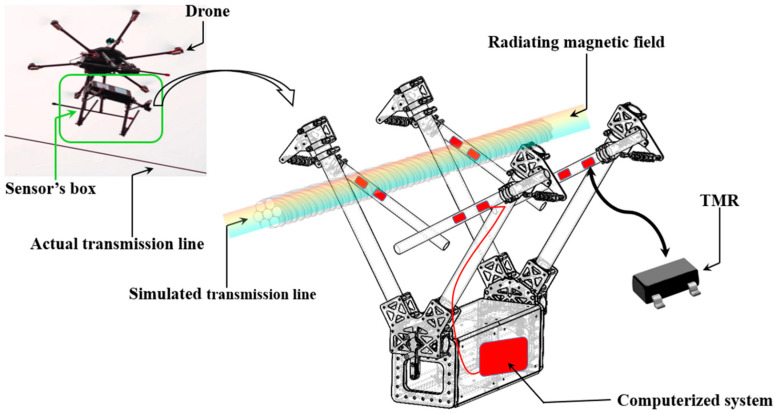
DNeD’s project overview [314].

In accordance with the review conducted in this manuscript, the Digitalisierte, rechtssichere und emissionsarme flugmobile Inspektion und Netzdatenerfassung mit automatisierten Drohnen (DNeD) project reflects an actual project that coincides with the concluded information [313]. DNeD aims to design an autonomous UAV that is able to inspect overhead Transmission Lines (TL) automatically and maneuver around them at a safe distance. The UAV should independently plan and execute the approach of flight/landing under prevailing conditions (e.g., cable routing). The UAV’s mission is to deploy its embedded sensor box on the transmission line. The sensor box in turn (i.e., payload) should allow for the remote monitoring of the electrical measurements (i.e., current and voltage) of the line. The data acquired from the TL, non-invasively by means of the deployed sensor box shown in Figure 12, should be intelligently able to be processed and visualized by a human operator at any time.

With the provided DNeD project’s description, this review serves as a straightforward informative background, allowing for the full realization of such a project. According to the investigated information in this paper, the DNeD can be decomposed into two major objects of research as follows: first, the drone with its relative avionics (SBCs/SoM with the correspondent sensors, communication/power modules, and algorithms for data processing). Second, the sensor box, constituting the payload with its total weight and architecture, as shown in Figure 12, and how it can affect the UAV’s flight path. The UAV’s full autonomy and obstacle (i.e., in this case, the TL) avoidance can be covered by the control subsystem reviewed in this manuscript: it is worthwhile to try the YOLOv8 algorithm in a powerful GPU-embedded SBC (e.g., NVIDIA Jetson) in order to detect the presence of a TL such that the UAV’s localization can be achieved through a set of georeferenced images, captured via an embedded camera. The entire UAV can be Li-I powered, with LoRa-enabled communication modules installed, mainly because of signals degradation near TL. In this case, the payload will be the sensor box, which is far from regular applications such as package delivery or military applications. This payload will have its boundaries set with an appropriate material type (i.e., electromagnetic permeability, rigidness, etc.) to ensure the stable remote monitoring of TL. Overall, the execution of the DNeD project with respect to the information provided in this manuscript is a worthy future project.

## 7. Conclusions

UAVs are complex electromechanical systems, rapidly evolving, and applicable in various fields. They represent a closed-loop interactive platform with five major subsystems (as concluded by this paper). For a stable UAV mission, each of the reviewed subsystems must be carefully engineered to fit at best compatibility with other subsystems. The overall findings can be compressed into one sequential mechanism: the control subsystem is the main decision-maker concerning the flight path (i.e., navigation), flight safety (i.e., collision avoidance), and flight mission (i.e., payload integration). The data input for the control subsystem is fed through the UAV-installed sensors (i.e., environmental, vision, position), and processed via the computing subsystem (i.e., SBCs, SoM). When needed, data can be externally communicated via single/multi communication modules (i.e., LoRa, BLE, Wi-Fi, LTE-M). The power subsystem eventually supplies the required energy of each of the subsystems. The choice of physical devices in each of the subsystems is a real challenge, which is mainly dependent on a UAV’s flight purpose.

Specifically concerning TL monitoring applications (e.g., the DNeD project), with an electrical sensor as the payload, the SBC is at best NVIDIA Jetson series-based (i.e., powerful GPU for TL identification/detection). LoRa-based communication modules, on the other hand (e.g., RN2483), would provide a good signal transmission with regard to electrical/magnetic noise emitted around the TL. An optimized Li-I would represent an optimum trade-off between safety and flight endurance. The sensor box itself is the required sensing equipment for TL monitoring/non-invasive measurements.

Eventually, the presented concrete lists of reviewed UAV’s avionics (i.e., ARM-based SoM, ARM/Samsung-based SBCs, LoRa modules (SX1278, HOPERF RFM95W-86852, etc.), BLE modules (nRF54H20, CC2650, etc.), sensors, power supplies, etc., make this review a handy tool for the hardware design of any other UAV mission purposes.

## Figures and Tables

**Table 1:** Assessment of vision-based UAV navigation methods.

Vision-Based Navigation for UAVs			
Advantages	Disadvantages	Challenges	Field of Application
✓ Informative scene data	✕ Complex environment structures reflect complexities in the navigation algorithm	Real-time processing requirements	Agriculture
✓ Anti-jamming ability	✕ Performance is impacted by adverse weather conditions	Integration with image-based sensing modalities	Surveillance
✓ Relatively high accuracy	✕ Vulnerable to visual illusions	Power consumption	Environmental monitoring

**Table 2:** Assessment of AI mathematical-based algorithms for UAV navigation.

		Mathematical-Based AI Algorithms		
Algorithm	Ref.	Performance	Efficiency	Contribution
PSO	[112]	High	Moderate	Non-feasible paths can be attained by means of an error factor
ACO	[113]	Moderate	High	Intra-/inter-colony yield a better convergence toward an optimum
GA	[114]	High	High	Chromosome decoding yields path navigation acknowledgment
DE	[115]	Moderate	High	Better convergence is achieved by means of selective mutations
GWO	[116]	High	High	Flexible algorithm hybridization with UAV navigation-based data

**Table 3:** Assessment of different sets of path planning and obstacle avoidance algorithms.

Algorithms Set	Working Mechanism
Sample-based	Divide the environment into a set of nodes. Connect nodes via “depth-first” search procedure. Begin the searching process for the optimal UAV route. Easy to implement and better work online.
Mathematical-based	Describe constraints (i.e., dynamic/kinematic) mathematically. Bind the cost function. Require high computational resources. Work better offline.
Multi-fusion	Integrate several algorithms. Save time. Appropriate to work online.
Bio-inspired	Heuristic-based. Excellent handling of complex unstructured constraints. Mutation is a key factor for path optimization. Appropriate to work offline.

**Table 4:** Assessment of the YOLOv6, YOLOv7, and YOLOv8 algorithms.

		YOLOvx-Algorithm Aspect		
Algorithm	Ref.	Working Mechanism	Additional Improvements	Performance
YOLOv6	[181,182]	Anchor-free. Decoupled head architecture (i.e., backbone: EfficientRep and neck: Rep-PAN). Two loss functions for classification/regression.	Knowledge distillation (i.e., teacher–student training model)	Achieves higher mean Average Precision (mAP) at different Frames Per Second (FPS) than its predecessors
YOLOv7	[183,184]	Extended-Efficient Layer Aggregation Network (E-ELAN) is implemented in its backbone. Compound model scaling. Module level re-parametrization.	Presents trainable Bag-of-Freebies	Improving accuracy simultaneously with maintained high detection speeds
YOLOv8	[185]	Backbone (CSPDarknet53) is modified with five times sampled input features. An enriched information flow is acquired by means of C2f (i.e., the number of bottlenecks). Feature maps are efficiently pooled via the Spatial Pyramid Proofing Fast (SPPF) module.	Dynamic task-aligned allocator	Positive and negative samples are specified by an anchor-free detection model

**Table 5:** Assessment of UAV SBCs.

SBC		Processor	RAM	Communication *	GPU	CPU Clock	Pros	Cons
Raspberry Pi 4		64-bit quad-core ARM	4 GB LPDDR4	Ethernet, USB, HDMI, Bluetooth, Wi-Fi, I2C, SPI, UART	Videocore VI	1.5 GHz	Upgradable RAM to 8 GB	Overheating
Odroid XU4		Samsung Exynos 5422 octa-core	2 GB LPDDR3	USB, Ethernet, HDMI, I2C, SPI, UART	Mali-T628 MP6	2 GHz	High processor performance	Incompatible with 3.3 V and 5 V accessories
NVIDIA Jetson	TX2	Dual-core NVIDIA Denver 2 64-bit; quad-core ARM Cortex A57	8 GB LPDDR4	Ethernet, USB, HDMI, UART, SPI, I2C, CAN	256-core NVIDIA Pascal	2 GHz	GPU acceleration	High power consumption
	Nano	Quad-core ARM Cortex A57	4 GB LPDDR4	Ethernet, USB, HDMI, SPI, I2C, UART, CAN	NVIDIA Maxwell	1.43 GHz	Good parallel processing	Overheating

* HDMI: High-Definition Multimedia Interface; I2C: Inter-Integrated Circuit; SPI: Serial Peripheral Interface; UART: Universal Asynchronous Receiver–Transmitter; CAN: Controller Area Network.

**Table 6:** Assessment of different UAV SoMs.

	SoM Brand			
Criteria	NXP I.MX8M	Rockchip RK3399	Qualcomm Snapdragon	STM32 *
Processor	ARM Cortex A53, A72	ARM Cortex A53, A72	ARM Qualcomm Kryo	ARM Cortex-M4
RAM	Up to 4 GB LPDDR4	Up to 4 GB LPDDR4	Up to 8 GB LPDDR4	Up to 640 kB SRAM
Main programming languages	C, C#, C++, Python, Java	C, C++, Python, Java	C, C#, C++, Kotlin, Java	C, C++, MicroPython
Programming structure	Sequential, concurrent, asynchronous, real-time	Sequential, concurrent, asynchronous, real-time	Sequential, concurrent, asynchronous, real-time	Sequential, concurrent, asynchronous, real-time
Embedded wireless communication	Wi-Fi, Bluetooth	Wi-Fi, Bluetooth	Wi-Fi, Bluetooth	-
Power consumption	Low	Moderate	Moderate	Very low
Supported temperature range	−40 °C to +105 °C	−40 °C to +80 °C	−40 °C to +105 °C	−40 °C to +125 °C
Outperforms in	Multimedia, industrial IoT	Multimedia, industrial IoT	AI, graphic processing, 5G	Real-time processing, embedded applications

* SRAM = Static Random-Access Memory.

**Table 7:** Assessment of different UAV’s communication modules.

		Characteristics						
			Range *					
Communication Technology	Module	Power Consumption	Indoor [km]	Outdoor [km]	Supported Frequency Ranges [Hz]	Max Data Rate (kbps)	RAM (Bytes)	Transmission Power [dBm]
LoRa	SX1278	Low	5–10	20	137–1020 MHz	300	256–512	20
	RN2483	Low	5–10	20	433;868;915 MHz	300	32 k	18
	HOPERF RFM95W-86852	Low	5–10	20	860–1020 MHz	300	256–512	20
Wi-Fi	ESP8266	Moderate	0.05–0.1	0.3	2.4 GHz	72	96–160	19
	ESP32	Moderate	0.05–0.1	0.3	2.4;5 GHz	150	520–320 k	19–20
	CC3000	Moderate	0.03	0.1	2.4 GHz	10	8 k	14
BLE	nRF54H20	Low	0.05–0.15	0.2–0.4	2.4 GHz	2	192–256	−40 to +8
	nRF54LI5	Low	0.05–0.15	0.2–0.4	2.4 GHz	2	192–256	−40 to +8
	CC2650	Low	0.05–0.15	0.2–0.4	2.4 GHz	2	20–80 k	−40 to +5
LTE-M	Quectel BG95-M3LGA	Low	-	-	LTE-M/NB-IoT/GSM/GPRS	588	32–64 M	23
	Telit ME310G1-WW	Low	-	-	LTE-M/NB-IoT	588	64 M	23

* The range of the LTE-M modules is dependent on network coverage. Other ranges are taken as the most probable averages.

**Table 8:** Assessment of this paper in regards with other similar publications.

Ref.	Criteria														
	Control			Computing		Communication				Sensory			Power		
	Navigation	Target Tracking	Payload Integration	SBCs	SoM	LoRa	Wi-Fi	BLE	LTE-M	Environmental	Vision	Position	Battery	PV–Batt	Gasoline–Batt
This work	✔	✔	✔	✔	✔	✔	✔	✔	✔	✔	✔	✔	✔	✔	✔
[305]	✔	✔	✔	✕	✕	✕	✕	✕	✕	✕	✕	✕	✕	✕	✕
[306]	✕	✕	✕	✕	✕	✕	✕	✕	✕	✕	✔	✔	✕	✕	✕
[307]	✔	✔	✔	✕	✕	✕	✕	✕	✕	✕	✔	✕	✕	✕	✕
[308]	✕	✕	✕	✕	✕	✕	✕	✕	✔	✕	✕	✕	✕	✕	✕
[309]	✕	✕	✕	✕	✕	✔	✔	✔	✔	✕	✕	✕	✕	✕	✕
[310]	✔	✔	✔	✕	✕	✕	✕	✕	✕	✔	✔	✕	✕	✕	✕

## Data Availability

The data are unavailable due to privacy or ethical restrictions.
